# Genetic Alterations in Essential Thrombocythemia Progression to Acute Myeloid Leukemia: A Case Series and Review of the Literature

**DOI:** 10.3389/fonc.2018.00032

**Published:** 2018-02-19

**Authors:** Jackline P. Ayres-Silva, Martin H. Bonamino, Maria E. Gouveia, Barbara C. R. Monte-Mor, Diego F. Coutinho, Adelmo H. Daumas, Cristiana Solza, Esteban Braggio, Ilana Renault Zalcberg

**Affiliations:** ^1^Bone Marrow Transplantation Unit, Specialized Laboratories, Laboratory of Molecular Biology, National Cancer Institute (INCa), Rio de Janeiro, Brazil; ^2^Programa de Carcinogênese Molecular, National Cancer Institute (INCa), Rio de Janeiro, Brazil; ^3^Vice-presidência de Pesquisa e Coleções Biológicas, Oswaldo Cruz Foundation, Rio de Janeiro, Brazil; ^4^Hematology Department, Chemotherapy Unit, Hospital Universitario Antonio Pedro – HUAP, Rio de Janeiro, Brazil; ^5^Hematology Unit, Hospital Universitario Pedro Ernesto – HUPE, Rio de Janeiro, Brazil; ^6^Department of Hematology and Oncology, Mayo Clinic, Scottsdale, AZ, United States

**Keywords:** essential thrombocythemia, secondary acute myeloid leukemia, array-based comparative genomic hybridization, whole exome sequencing, myeloproliferative neoplasms, +2p

## Abstract

The genetic events associated with transformation of myeloproliferative neoplasms (MPNs) to secondary acute myeloid leukemia (sAML), particularly in the subgroup of essential thrombocythemia (ET) patients, remain incompletely understood. Deep studies using high-throughput methods might lead to a better understanding of genetic landscape of ET patients who transformed to sAML. We performed array-based comparative genomic hybridization (aCGH) and whole exome sequencing (WES) to analyze paired samples from ET and sAML phases. We investigated five patients with previous history of MPN, which four had initial diagnosis of ET (one case harboring *JAK2* p.Val617Phe and the remaining three *CALR* type II p.Lys385fs*47), and one was diagnosed with MPN/myelodysplastic syndrome with thrombocytosis (*SF3B1* p.Lys700Glu). All were homogeneously treated with hydroxyurea, but subsequently transformed to sAML (mean time of 6 years/median of 4 years to transformation). Two of them have chromosomal abnormalities, and both acquire 2p gain and 5q deletion at sAML stage. The molecular mechanisms associated with leukemic progression in MPN patients are not clear. Our WES data showed *TP53* alterations recurrently observed as mutations (missense and frameshift) and monoallelic loss. On the other hand, aCGH showed novel chromosome abnormalities (+2p and del5q) potentially associated with disease progression. The results reported here add valuable information to the still fragmented molecular basis of ET to sAML evolution. Further studies are necessary to identify minimal deleted/amplified region and genes relevant to sAML transformation.

## Introduction

Essential thrombocythemia (ET) is a myeloproliferative neoplasm (MPN) derived from clonal expansion of hematopoietic progenitor cells carrying a mutation in the cytokine receptor/JAK2-signaling pathway (*JAK2, CALR*, or *MPL*). ET patients present platelet overproduction, bone marrow biopsy with enlarged megakaryocytes with hyperlobulated nuclei, with no left shift to granulopoiesis or erythropoiesis, and very rarely reticulin fibers. Other symptoms include transient ischemic attacks, ocular migraine, erythromelalgia, acquired von Willebrand disease, and pseudohyperkalemia due to extreme thrombocytosis, arterial or venous thrombosis (less common), and transformation to bone marrow failure such as myelofibrosis. Around 1 to 5–6% evolve to secondary acute myeloid leukemia (sAML), which has dismal prognosis with most of the patients dying within few months (1–3).

Regarding the MPN/AML transformation, mutations in genes encoding epigenetics modifiers such as *ASXL1, IDH1, IDH2, EZH2*, and *TET2* are associated with nearly 30% of secondary leukemia transformations and *de novo* acute myeloid leukemia (4, 5). Mutations in *IDH1* and *IDH2* are observed in around 15% of AML, and specifically detected around 20–30% of sAML samples from MPN patients (5, 6). *ASXL1, EZH2*, and *TET2* are the second largest mutated subgroup of genes in patients with AML and also compose a high-risk subcategory in myeloproliferative and myelodysplasic neoplasms. These mutations are acquired earlier in the disease and have prognostic values (5). Genetic changes and clonal evolution associated with ET to sAML progression remain incompletely understood in nearly 70% of patients. Genome wide analysis is an useful tool to understand the molecular events underlying sAML transformation in those cases.

Here, we describe five MPN patients diagnosed with thrombocytosis characterized by high platelet counts (from 623k × 10^9^/L to 2,395k × 10^9^/L), hyperplasia, and enlarged megakaryocytes with hyperlobulated nuclei at bone marrow biopsy. Physical exam and imaging analysis showed no splenomegalia during follow-up. All patients were homogeneously treated with hydroxyurea and evolved to sAML. Peripheral blood (PB) molecular screening showed three patients with CALR type II, one *JAK2* p.Val617Phe, and one with *SF3B1* p.Lys700Glu. MPN and sAML samples from all five patients were analyzed by array-based comparative genomic hybridization (aCGH). Three of these patients showed molecular alterations by aCGH and were further investigated by whole exome sequencing (WES).

## Materials and Methods

This study was carried out in accordance with the recommendations of the “Institutional Ethics Committee (Brazilian National Institute of Cancer)” with written informed consent in accordance with the Declaration of Helsinki. The clinical data provided in the current case report contain no personal health information, identifier or personal feature, to protect participants’ rights. Written informed consent was obtained from some of the participant close relatives for the publication of this case report. Other participants have from 4 to 8 years since decease and clinical staff has lost follow-up with patient’s relatives, making it impossible to get an informed consent for publication.

### Patients

**Figure 1:**
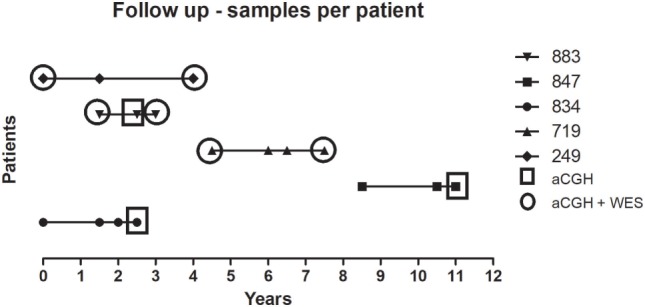
Longitudinal analysis of uniformly treated MPN cases. Five cases were analyzed by aCGH and WES before and after therapy. The horizontal axis represents the timeline (in years) of sample collection. Each case is presented in the right legend. Squares are showing samples analyzed only by aCGH and circles by the two methodologies applied. aCGH, array-based comparative genomic hybridization; MPN, myeloproliferative neoplasm; WES, whole exome sequencing.

The reported patients are part of a retrospective multicenter study (Hospital Universitário Pedro Ernesto-HUPE/UERJ and Hospital Universitário Antônio Pedro-HUAP/UFF) carried out between January 2007 and August 2015. Among the 158 MPN patients followed up until death, there were 38 with Polycythemia Vera (PV), 91 with ET, and 29 with primary Myelofibrosis (PMF). Six patients transformed to secondary myelofibrosis (secMF) and eight to sAML. From those eight with sAML, three patients were excluded for further analysis because paired samples from MPN and transformation phases were lacking. Five patients were selected for further genomic analysis, and time points analyzed are summarized in Figure 1. Four patients have initial diagnosis of ET and one of MPN/myelodysplastic syndrome (MDS) and clinical and laboratorial features are summarized in Table 1. All the patients were tested for *JAK2V617F, CALR*, and *MPL* mutation, and they were classified by the World Health Organization criteria (7).

**Table 1:** Clinical and laboratorial features of patients who progressed to sAML.

	Diagnosis	sAML	*p*
Gender (female/male)	5:0	5:0	
Age (years), median (range)	59 (41–63)	62 (51–70)	0.0625
Follow-up (days), median (range)	1,527 (978-4171)	56 (31–299)	0.0625

**Hematological features**			
Hematocrit (%)	35.1 (23–45)	24.4 (20.2–27.6)	0.0625
Hemoglobin (g/dL)	11.9 (7.1–14.0)	8.0 (6.5–9.0)	0.0625
WBC (10^9^/L)	8.9 (5.3–48.9)	17.8 (7.9–102.2)	0.0625
Neutrophils (10^9^/L)	5.2 (3.4–18.5)	8.1 (2.4–42.9)	0.6250
Lymphocytes (10^9^/L)	2.0 (1.3–8.9)	2.4 (0.9–9.1)	>0.9999
Monocytes (10^9^/L)	0.8 (0.3–1.7)	2.1 (0.07–30.6)	0.25
Platelets (10^9^/L)	889 (623–2,395)	196 (71–354)	0.0625
Blasts (%)	0	34 (27–44)	0.1250

**Events during follow-up^a^**			
Weight loss	1		
Hemorrhage	2		
Thrombosis	1		
Paresthesia	0		
Pruritus	0		
Splenomegaly	0		
Hepatomegaly	0		

*^a^Events occurred since MPN diagnosis*.

*MPN, myeloproliferative neoplasm; sAML, secondary acute myeloid leukemia*.

### Samples

#### DNA Extraction

Peripheral blood samples were collected into EDTA and processed within 24 h of collection in each case. DNA extraction was performed from granulocytes after submitting the blood sample to Ficoll-Hypaque^®^ gradient. The red cells were removed by hypotonic lysis solution. Cell pellets were resuspended in DNAzol^®^ (Invitrogen), and DNA was extracted following the manufacturer’s protocol. Samples cleanup was done using puregene kit (Qiagen) or phenol:chloroform:isoamyl alcohol (25:24:1) protocol.

#### Mutation Screening

All genomic DNA samples were tested for V617F allele by allelic specific PCR (ASO PCR) as previously reported (8) with minor modifications (9) and also screened for the following mutations: *JAK2* exon 12, *MPL* exon 10, *CALR* mutations, *SF3B1* exons 14 and 15, *IDH1/2* exon 4, and *TET2* exons 3 and 9. *CALR* indel analysis was performed by PCR with fluorescent primers followed by fragment analysis and by Sanger sequencing (10). *JAK2* exon 12, *MPL, TET2, SF3B1*, and *IDH1* and *IDH2* mutations were examined by Sanger sequencing with BigDye Terminator v3.1 kit as described by the manufacturer. We used the following primers to amplify *JAK2* exon 12 (F—5′ CTCCTCTTTGGAGCAATTCA 3′/R–5′ GAGAACTTGGGAGTTGCGATA 3′), *MPL* gene (F—5′ TCCTAGCCTGGATCTCCTTGG 3′/R—5′ CACAGAGCGAACCAAGAATGCC 3′). *TET2* mutations were evaluated with primers described by Martínez-Avilés et al. (11), *SF3B1* as described by Cui et al. (12), *IDH1/2* following Wagner et al. (13) and *TP53* by applying IARC database-based method (14).

#### Array-Based Comparative Genomic Hybridization

Array-based comparative genomic hybridization was performed in five cases using the Human Genome 244A and the Sureprint G3 microarrays (Agilent Technologies). Genomic DNA from PB granulocytes was used. The digestion, labeling, and hybridization steps were done as previously described in Ref. (15, 16). Briefly, 1.2 μg of tumor and reference DNA were independently fragmented with Bovine DNaseI (Ambion, Austin, TX, USA) for 12 min at room temperature. DNA samples from a pool of nine human, female, lymphoblastoid cell lines from the Coriell repository were used as the normal reference in the hybridization experiments. Tumor samples were labeled with Alexa 5 dye, and the normal reference was labeled with Alexa 3 dye. Labeled reactions were cleaned up and hybridized at 65°C for 40 h. Microarrays were scanned in a DNA Microarray Scanner (Agilent), and features were extracted with Feature Extraction software (Agilent) version 10.7.3.1 (Agilent Technologies). Extracted data were imported, and log2 ratios were analyzed using Genomic Workbench software version 5.0.14 (Agilent Technologies). Copy number aberrations were calculated using ADM-1 algorithm with a 6.0 threshold, 5 probe filter, and log2 ratio above/below ±0.25. To identify and eliminate the germline copy number variations (CNVs) from the study, we created a CNV database including the recent higher-resolution copy number (using platform SNP6.0) and sequencing studies available in The Center for Applied Genomics data portal^1^ as well as our findings in 10 HapMap samples run by Sureprint G3 arrays (16).

#### Paired-End WES

Genomic DNA from PB granulocytes from each sample was sheared and used for the construction of paired-end sequencing library as described in the protocol provided by Illumina. The exome capture was done using the Sure Select 50Mb Exome Enrichment kit (Agilent) following the manufacturer’s instructions. Next, 100 bp paired-end DNA libraries were prepared, and three samples were run per lane in the HiSeq2000 sequencer (Illumina). An automated workflow for exome-seq data analysis was developed. First, 100 bp paired-end reads were aligned to human genome hg19 using Novoalign (Novocraft Technologies, Malaysia). Quality of sequencing chemistry was evaluated using FastQC.^2^ Realignment and recalibration were done using Best Practice Variant Detection v3 recommendations implemented in the GATK.^3^ After alignment, PCR duplication rates and percent reads mapped on target were used to assess the quality of the data. Somatic single nucleotide variants (SNVs) were genotyped using SomaticSniper(17), whereas insertions and deletions were called by GATK Somatic Indel Detector. Each variant in coding regions was functionally annotated by snpEFF^4^ and PolyPhen-2 (18) to predict biological effects. The variants were annotated using our TREAT workflow (19) whether the gene is associated with disease or phenotypes and any associated pathways. We removed variants found in the 1,000 genomes,^5^ the Exome Variant Server NHLBI Gene Ontology (GO) Exome Sequencing Project (Seattle, WA, USA),^6^ and the BGI—Danish Sequencing Project. In addition, we removed variants present in dbSNP data set unless these mutations were also present in the COSMIC database. Variants with read depth less than 10× were excluded from further analysis. Additional germline variants were excluded from analysis after comparing with 25 non-tumoral patient samples. Finally, non-synonymous and non-exonic variants of significant interest were visually inspected using IGV (20).

#### Clustering Analysis

Clustering analysis was run using clValid package (21) from R software,^7^ using all somatic mutations (intronic and exonic) that showed more than 50× coverage in the tumor samples. To define the number of clones presented, we combine data from nine clustering models (hierarchical clustering, k-means, DIANA, PAM, CLARA, FANNY, SOM, Expectation-Maximization, SOTA) and elected as the number of clusters presented in each patient the result recurrently found in most of nine models analyzed. Other algorithms were also used: kohonen and mclust for Expectation-Maximization algorithm. Internal validation was analyzed by connectivity, Dunn and Silhouette. We also used a fpc package from R software and run a prediction strength and nselectboot function for selection of the number of clusters *via* bootstrap and computes the prediction strength of a clustering of a data set into different numbers of components.

#### Gene Set Enrichment Analysis

Enrichment analysis on gene sets of functional gene classes or ontology terms was performed using the analysis tool provided by Database for Annotation, Visualization and Integrated Discovery^8^ (22, 23) maintained by National Institute of Allergy and Infectious Diseases (NIH) using Protein Information Resource, Kyoto Encyclopedia of Genes and Genomes, or GO Consortium databases to execute enrichment examination. We analyzed genes contained in each chromosome abnormality region (gain or loss) or genes set from each patient containing somatic mutations [by point or insertions–deletions (INDELS)] detected by WES.

#### Statistical Analysis

Hematological data were analyzed using two-sided Wilcoxon signed-rank test with *p* = 0.05 using Prism 6 (Graphpad, La Jolla, CA, USA).

## Results

### Clinical Presentation

Clinical and hematological data are summarized in Table 1. All the patients were female, and median follow–up time for those five patients included were 1,527 days (range, 978–4,171 days) from diagnosis until transformation and 56 days (range, 31–299 days) during sAML. Weight loss, hemorrhage, and thrombosis were the main clinical presentations observed during follow-up. All patients were screened for mutations previously described to be more frequently found upon transformation such *IDH1/IDH2* (exon 4) and *TET2* (exon 3) but were absent at MPN and sAML samples. Other clinical relevant features from each patient are described as follows:

**Case 1** (UPN834)**:** A patient diagnosed with ET, CALR type II positive (p.Lys385fs*47) with bone marrow biopsy at diagnosis showing proliferation of all lineages with increased number of mature megakaryocytes. The patient was treated with hydroxyurea, and no adverse events or thrombotic events were reported during follow-up. Three years after diagnosis, a new bone marrow biopsy was performed, which showed 44% blasts and presence of megakaryoblasts. At that time, the patient was still JAK2 p.Val617Phe negative, CALR type II positive and aCGH analysis in the sAML sample showed no chromosome abnormalities.
**Case 2** (UPN847)**:** A patient was diagnosed with ET, showed CALR type II mutation (p.Lys385fs*47), with bone marrow biopsy at diagnosis showing megakaryocyte proliferation with hyperplasia and absence of fibrosis. The patient was treated with hydroxyurea. A new bone marrow biopsy was performed 12 years after diagnosis and revealed atypic cells and presence of micromegakaryocytes. No chromosome abnormalities were observed in the granulocyte fraction at sAML blood sample by aCGH.
**Case 3** (UPN719)**:** A patient was diagnosed with ET and was JAK2 p.Val617Phe positive. The patient was treated with hydroxyurea, but during follow-up presented a thrombosis in left lower limb, epistaxis, and weight loss. A new bone marrow biopsy was performed 7.5 years after diagnostic and showed hyperplasia of megacaryocytes, elevated myeloid/erythroid ratio, and a presence of myeloblasts. Paired aCGH analysis of initial diagnosis and sAML samples evidenced eight chromosome abnormalities at chromosomes 2, 3, 5, 6, and 8 at progression (Table 2). WES analysis of these same samples showed 171 single-nucleotide variants/INDELS (SNV/INDELS) with variant allele frequencies (VAFs) ratio higher than 1.5 between the two samples analyzed (Table S1 in Supplementary Material).
**Case 4** (UPN883)**:** A patient was diagnosed with ET, CALR type II (p.Lys385fs*47) positive with bone marrow biopsy showing bizarre megakaryocytes, hypocellularity in the myeloid and erythroid lineages, and grade I fibrosis. The patient was treated with hydroxyurea and showed no adverse events during follow-up. Since diagnosis, the patient remained with high platelet counts, which required unusual hydroxyurea doses of 2–3 g/day for more than 2/3 of treatment (1,200 days). After 2 years, cytopenia forced the reduction of hydroxyurea to 0.5 g/day, until the leukemic transformation, about 140 days after the onset of cytopenia. Three years after diagnosis new bone marrow biopsy was performed and showed myeloblasts. aCGH analysis of ET and sAML samples showed 10 chromosome abnormalities found at both chronic and acute phases and six other abnormalities found only at sAML progression (Table 2). WES analysis of two paired samples revealed 211 SNV/INDELS with chronic/sAML VAF ratio at least more than 1.5 (Table S1 in Supplementary Material).
**Case 5** (UPN249)**:** A patient with molecular profile showing SF3B1 (p.Lys700Glu) positive. Bone marrow biopsy could not evidence ring sideroblats and was diagnosed with MPN/MDS. The patient was treated with hydroxyurea and had no adverse events during follow-up. A new bone marrow biopsy 4.7 years after diagnosis showed myeloblasts. Paired ET/sAML analysis by aCGH detected one chromosome abnormality found in both samples (12q24.31) and five other abnormalities found upon leukemic transformation (Table 2). WES screening identified 206 differentials SNV/INDELS with VAF ratio chronic/sAML at least more than 1.5 (Table S1 in Supplementary Material).

**Table 2:** Comparative list of chromosome abnormalities found since MPN phase, showing differences in chromosome gain and losses in the two samples analyzed per patient.

Patient	Chronic					sAML				
	Chromosome position	Cytoband	Abnormality type	Size (Mb)	Genes	Chromosome position	Cytoband	Abnormality type	Size (Mb)	Genes
249	chr12:120136566-121217263	q24.31	Loss	1.08	16	chr12:120136566-121217263	q24.31	Loss	1.08	16
						chr02:20341-9902169	p25.3 - p25.1	Loss	9.88	35
						chr09:24744409-44167273	p21.3 - p11.2	Loss	19.42	138
						chr09:70233135-140145683	q13 - q34.3	Loss	69.91	580
						chr18:29457213-29542717	q12.1	Loss	0.09	1
						chr18:39765064-76103196	q12.3 - q23	Loss	36.34	136

719						chr02:20341-55630604	p25.3 - p16.1	Gain	55.61	287
						chr03:140569811-196800093	q23 - q29	Gain	56.23	272
						chr05:88523068-180644810	q14.3 - q35.3	Loss	92.12	576
						chr06:97634-26811016	p25.3 - p22.1	Loss	26.71	170
						chr06:27753009-55311370	p22.1 - p12.1	Gain	27.56	446
						chr08:111851857-112916955	q23.2 - q23.3	Gain	1.07	0
						chr08:122563718-142239028	q24.13 - q24.3	Gain	19.68	64
						chr17:1427745-2751645	p13.3	Gain	1.32	25

883	chr01:52157850-53158142	p32.3	Loss	1.00	15	chr01:52157850-53158142	p32.3	Loss	1.00	15
	chr01:5566063-8088072	p36.31 - p36.23	Loss	2.52	27	chr01:5566063-8088072	p36.31 - p36.23	Loss	2.52	27
	chr03:47209546-49760081	p21.31	Loss	2.55	63	chr03:46682145-50640965	p21.31	Loss	3.96	105
	chr03:37196477-38045070	p22.2	Loss	0.85	6	chr03:37196477-38045070	p22.2	Loss	0.85	6
	chr03:12875798-19031033	p25.1 - p24.3	Loss	6.16	35	chr03:12875798-19031033	p25.1 - p24.3	Loss	6.16	35
	chr03:130110062-133080818	q21.3 - q22.1	Loss	2.97	34	chr03:130110062-133080818	q21.3 - q22.1	Loss	2.97	34
	chr07:100510862-101901101	q22.1	Loss	1.39	21	chr07:99897634-101901101	q22.1	Loss	2.00	43
	chr13:22693488-23192438	q12.12	Gain	0.50	3	chr13:22693488-23192438	q12.12	Gain	0.50	3
	chr17:2253374-7618837	p13.3 - p13.1	Loss	5.37	148	chr17:1770937-8171731	p13.3 - p13.1	Loss	6.40	185
	chr17:26023960-27839678	q11.2	Loss	1.82	23	chr17:26023960-27839678	q11.2	Loss	1.82	23
						chr02:29193-71490513	p25.3 - p13.3	Gain	71.46	372
						chr05:127521614-180644810	q23.3 - q35.3	Loss	53.12	468
						chr07:131473980-131634667	q32.3	Loss	0.16	1
						chr10:4974594-12038306	p15.1 - p14	Loss	7.06	36
						chr12:120665448-120755495	q24.31	Loss	0.09	4
						chr17:68792353-78638467	q25.1 - q25.3	Loss	9.85	199

*Human assembly—February 2009 GRCh37/hg19*.

*MPN, myeloproliferative neoplasm; sAML, secondary acute myeloid leukemia*.

### Genomic Analysis

ET transformation to sAML is a rare event. In our 91 ET patients cohort, we observed five cases of sAML transformation reported herein.

**Figure 2:**
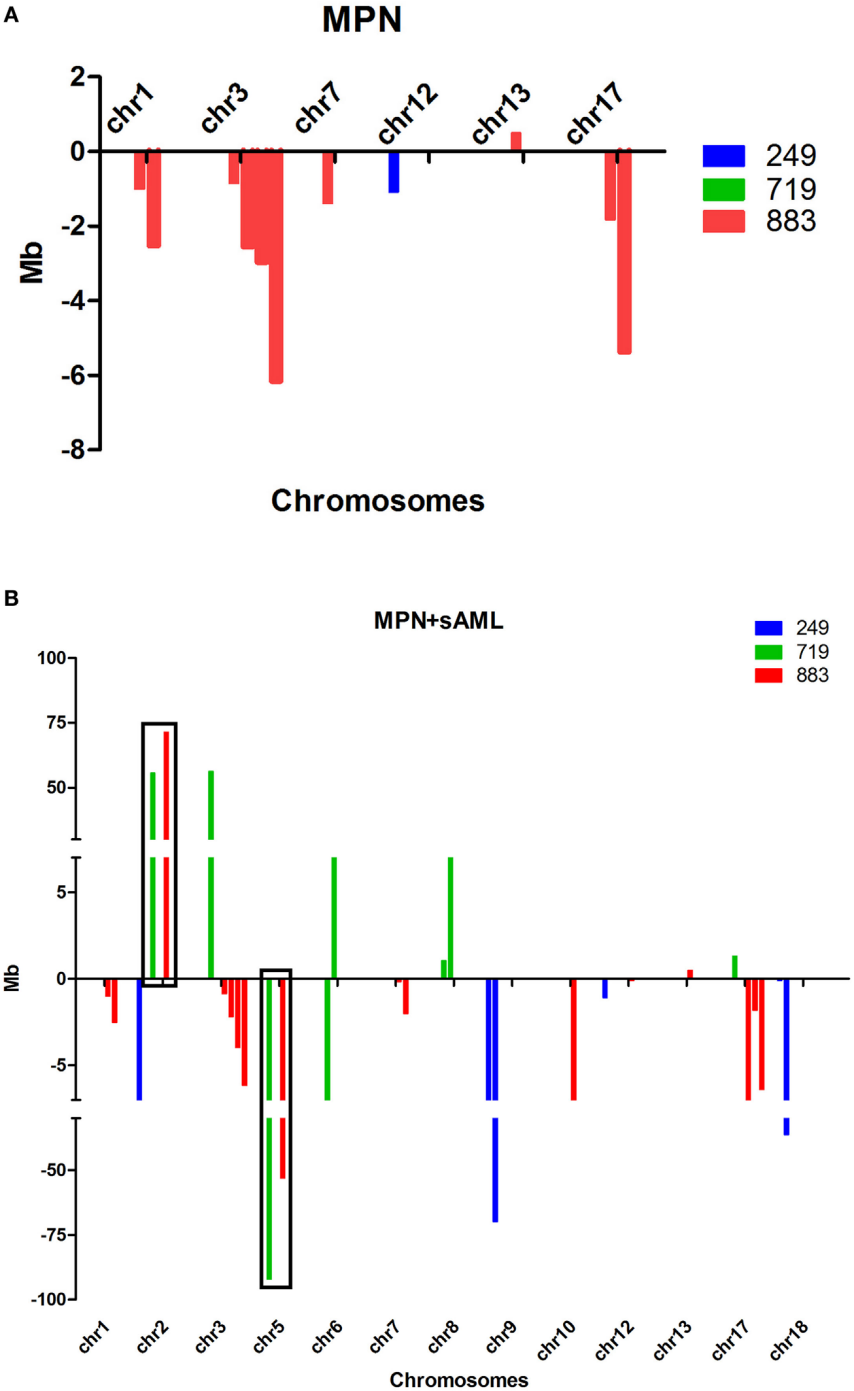
**(A,B)** Size and type of the abnormalities found at chronic phase **(A)** and sAML **(B)** samples from three patients analyzed by aCGH. Abnormalities found at chronic phase remained until sAML. Gains are represented above and losses below X-axis; note that there are more abnormalities found at sAML, and they are larger compared to chronic phase. aCGH, array-based comparative genomic hybridization; chr, chromosome; MPN, myeloproliferative neoplasms; sAML, secondary acute myeloid leukemia.

Comprehensive genomic characterization of paired samples by aCGH identified two common genetic abnormalities—gain of 2p and deletion of 5q—not identified in initial ET but present in the sAML phase, suggesting that they were acquired during disease progression (Table 2; Figures 2A,B). The common deleted region in chromosome five covers cytobands q23.3–q35.3 and the 2p minimal gain region comprised cytobands p13.3–p25.3.

**Figure 3:**
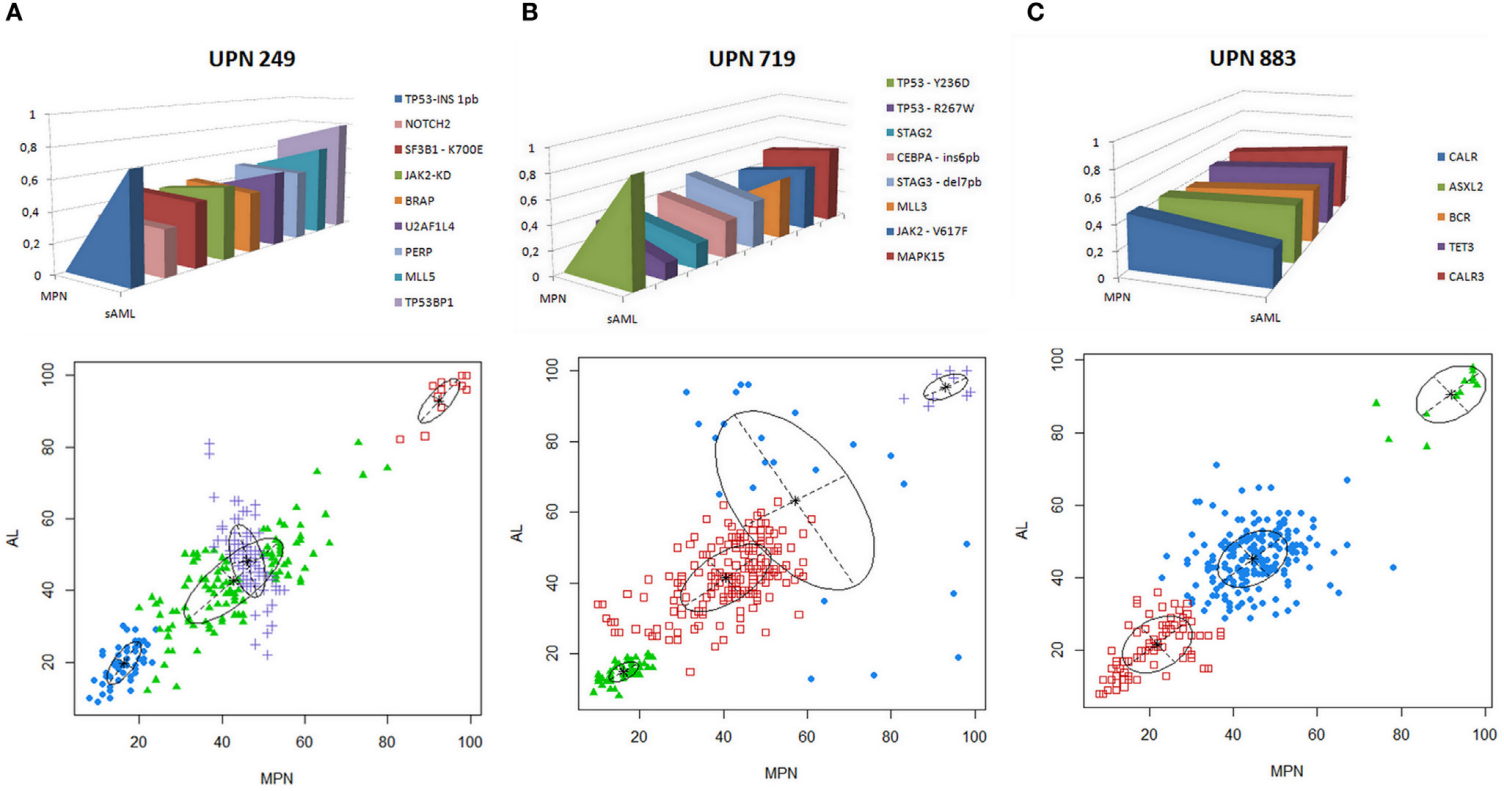
**(A–C)** Genomic landscape of thrombocythosis (ET and MPN/MDS) that progressed to sAML. VAF of candidate driver mutations or paralogous genes already associated with MPN phenotype in each patient analyzed are depicted in upper panels. Model of clonal evolution of MPN to sAML showing average VAF of each subclone in the stages analyzed. Clonal evolution complexity showing VAF of mutations found in each clone per patient in MPN (abcissa) and sAML (ordinate) stages is depicted in the lower panels. VAF plots were generated by mclust after running fpc and nselectboot functions. ET, essential thrombocythemia; MDS, myelodysplastic syndrome; MPN, myeloproliferative neoplasm; sAML, secondary acute myeloid leukemia.; UPN, unique patient number; VAFs, variant allele frequencies.

The three patients with chromosomal abnormalities were subsequently analyzed by WES. Mutations in paralogous genes already associated with MPN phenotype such as *CALR3, ASXL2*, and *TET3* were found augmented at sAML sample from ET patient positive for *CALR* (p.Lys385fs*47) (Figure 3C; Table S1 in Supplementary Material). A novel *JAK2* mutation p.Arg1063Cys was found at MPN/MDS patient who carries a *SF3B1* p.Lys700Glu since the first sample analyzed.

*TP53* alterations were recurrently observed with mutations in two cases (UPN719 and UPN249) and monoallelic loss (UPN883). UPN719 was characterized by the presence of *JAK2* p.Val617Phe and *TP53* p.Arg267Trp at initial ET stage and subsequent acquisition of *TP53* p.Tyr236Asp at sAML phase. UPN249 had a *SF3B1* p.Lys700Glu at chronic phase and subsequently acquired a *TP53* p.Leu106ArgfsTer25 in the progression to sAML. UPN883 had a monoallelic loss of *TP53* and a *CALR* p.Lys385fs*47 (Figures 3A–C).

The analysis of clonal architecture showed a founding dominant clone and the subsequent rise of a minor genetic subclone growing overtime in all the three cases analyzed (Figures 3A–C). No recurrent gene mutations were found among patients and samples analyzed, besides the *TP53* and *JAK2* alterations aforementioned (Table S1 in Supplementary Material).

## Discussion

Genomic studies evaluating serial samples of MPNs are few (24, 25), especially in the subgroup of ET (26, 27). Our work is one of the few reports associating aCGH and WES data in sequential paired samples from ET diagnosis to sAML progression.

Among the identified chromosomal alterations, we could identify +2p and −5q only at sAML samples. Chr2 gain is a novel genetic change described in the subgroup of MPN patients. Whether +2p and −5q abnormalities are derived from a pre-existing MPN small subclone or acquired in the sAML transformation is still a matter of debate, particularly in ET, where very few reports describing 2p gain (28) and 5q deletions associated with MPN exists (Table 3). Other possibility is that chromosome alterations are present but at low frequency, thus undetectable by the methods used in our approach. In contrast, −5q MDS represents a well-known subgroup with cellular and molecular mechanisms well understood, in contrast to MPN, which requires further studies.

**Table 3:** Comparative studies showing previous reports in +2p and del5q in myeloproliferative and myelodysplasia neoplasms.

Total cases studied	Diseases studied	Methodology	Paired sample?	sAML transformation	+2p^a^	5q (del)^a^	Reference
151	45 PV; **45 ET**; 47 PMF; 14 sMF	250k SNP-a	No	No	–	del 5q11-q13 (sMF)	Stegelmann et al. (29)
16	7 PV; **3 ET**; 5 PMF; 1 RARS-t	Cytogenetics	Yes	Yes	–	No	Beer et al. (30)
71	**13 ET**; 32 PMF; 26 PV	aCGH-44k and/or 105k and classic cytogenetic	No	No	–	Not specified	Tefferi et al. (31)
402	**ET**	Classic cytogenetic	28/402 had multiple samples analyzed	Yes	+2 (ET-sAML)	del(5)(q15q33) (chronic)/del(5)(q)del(9)(q) (sAML)	Gangat et al. (28)
148 pcts = 88 MPN; 71 MPN–sAML (11 paired samples)	38 PV + 2 sAML; **49 ET + 1 sAML**; 50 PMF + 8 sAML	SNP-a 50k and/or 250k and classic cytogenetic	11 (2 PV; 1 ET; 8 MPF)	Yes	add(2)(p22.3) (PV); add(2)(25pterp21) (PV); + 2 (PMF); add(2)(q24.3) (PMF)	del(5)(q11.2qter) (PV); del(5)(q14.1qter) (PV); del (5) (q23.1) (ET); del (5) (q) (ET); del (5) (q22.1 qter) (ET); del (5) (q14.3q33.3) (ET)	Thoennissen et al. (32)
23 Del5q	14 PMF; 2 PV; **1 ET; 1 ET–MF**; 1 SM; 4 MPN-U	Classic cytogenetic	No	No	–	Yes	Santana-Davila et al. (33)
358 del 5q	**25 MPN**; MDS; AML; PCPD; ALL; PCPD-MDS; MDS/MPN; LYMPHOMA	Classic cytogenetic	Not specified	Not specified	–	Yes	Santana-Davila et al. (34)
38	8 MPN; 30 MPN/MDS (non-classic)	250k SNP-a and classic cytogenetic	Yes	Yes—5 pcts/2 MPN; 3 MPN/MDS-U	–	del(5)(q12q33) (MPN/MDS-U) (chronic); del(5)(q13q33) (MPN/MDS-U) (chronic); del(5)(q13q33) (MPN/MDS-U) (chronic); del(5)(q32), del(5)(q13.3), del(5)(q31.2) (MPN/MDS-U) (sAML); del(5)(q21.3q33.3) (PV-sMF) (chronic)	Gondek et al. (35)
143 del 5q	88 MDS 5q; 13 RAEB-1; 9 RAEB2; 4 MDS-U; 2 CMML; 1 t-MDS; 4 PV; **3 ET**; 3 PMF; 1 MPN-U; 1 SM; 14 AML	Classic cytogenetics; molecular screening for JAK2 V617F and exon14; MPL;IDH	No	Yes—some	–	Yes—not specified	Patnaik et al. (36)
1		Classic cytogenetics	Yes	Yes	–	Yes—not specified	Rashidi et al. (27)

*aCGH, array-based comparative genomic hybridization; AML, acute myeloid leukemia; CMML, chronic myelomonocytic leukemia; ET, essential thrombocythemia; MDS, myelodysplastic syndromes; MDS-U, myelodisplastic syndromes unclassified; MPN, myeloproliferative neoplasms; MPN-U, myeloproliferative unclassified; PCPD, plasma cell proliferative disorder; PMF, primary myelofibrosis; PV, polycythemia vera; RAEB, refractory anemia with excess blasts; RARS-t, refractory anemia with ring sideroblast and thrombocytosis; sAML, secondary acute myeloid leukemia; SM, systemic mastocytosis; sMF, secondary myelofibrosis; t-MDS, therapy-related myelodysplastic syndrome*.

*^a^Each abnormality represents one patient studied, with diagnosis in parentheses*.

At MPN, we observed a cytokine cluster in the minimal 5q deleted genomic region compassing nine genes (*IL4, CSF2, TSLP, IL3, IL5, IL9, IL13, IL12B*, and *SPRY4*) that are part of the JAK-STAT signaling pathway associated with proliferation and differentiation of the hematopoietic compartment. The 2p minimal gain region includes known regulators of epigenetic processes such as *DNMT3A, ASXL2 e DPY30*. There are few reports showing chromosome 2 gain in MPN cases, and only one study showing this alteration in ET (28). A comparative analysis of published data describing alterations in this locus is presented in Table 3.

Genomic abnormalities targeting cytokines and cytokine receptors clusters are described in several types of tumors, such as loss of chromosome 4 and the subsequent decreased IL15 expression in colorectal cancer (37). The potential role of cytokine cluster deletions in MPNs depends on the description of such molecular phenomena in larger cohorts and on mechanistic studies on how these deletions can impact the leukemic niche and/or the sAML transformation process.

We identified a novel *JAK2* mutation at kinase domain that is predicted to result in STAT5 activation, as suggested by a previous study that showed a mutation in the same residue, *JAK2* p.Arg1063His, associated with STAT5 phosphorylation and increase of CFU-E formation, in addition to *in silico* modeling, which predicted a facilitation of the active conformation of JH1 (38).

Our work reinforces the relevance of *TP53* mutations in sAML progression and supports the previous descriptions that JAK2-negative patients can also acquire *TP53* mutations (39, 40). TP53 mutations in AML occurs around 10–20% and is attributed to poor prognosis. In recent years, diagnosis of TP53 dysfunction is growing as therapeutics drugs to target TP53 mutations is increasing and getting promising results (41, 42). Association of TP53 loss with 5q haploinsufficiency in mice promoting myeloid leukemia was observed, and studies associating other abnormalities such as +2p need further attention (43).

Clonal analysis showed the molecular heterogeneity among ET patients, as no recurrent gene mutation was found. Even comparing our data with other WES findings, we could not find any recurrent gene mutation, corroborating Engle et al. (25) findings describing that most mutations found in a patient with secondary myelofibrosis who progressed to sAML are passenger mutations, with a clonal architecture similar to the ones described in our patients.

## Conclusion

Secondary Acute Myeloid Leukemia progression is a rare event, especially in ET, with incidence rate of less than 5%. Because ET is the more indolent MPN, with a long natural disease history, studies with paired sample analysis are scarce. Genomic analysis of paired samples, as reported here, adds valuable information to understand the molecular basis of sAML transformation. Our data showed chromosome abnormalities potentially associated with disease progression not previously described, and those abnormalities (+2p and del5q) have common regions that should be further screened.

## Ethics Statement

This study was carried out in accordance with the recommendations of the Institutional Ethics Committee (Brazilian National Institute of Cancer), with written informed consent in accordance with the Declaration of Helsinki. The clinical data provided in the current case report contains no personal health information, identifier, or personal feature to protect participants’ rights. Written informed consent was obtained from some of the participant close relatives for the publication of this case report. Other participants have from 4 to 8 years since decease and clinical staff has lost follow-up with patient’s relatives, making it impossible to get an informed consent for publication.

## Author Contributions

JA-S, MB, IZ, and EB designed the study. JA-S, BM-M, MB, IZ, and EB analyzed molecular data and wrote the manuscript. JA-S and DC performed and developed molecular assays. MG, AD, and CS took care of the patients, performed clinical review of the cases, and contributed to the interpretation of data. All authors made a substantial contribution, reviewed, and approved the final version of the manuscript. All the authors are in agreement to be accountable for all aspects of the work in ensuring that questions related to the accuracy or integrity of any part of the work are appropriately investigated and resolved.

## Conflict of Interest Statement

The authors declare that the research was conducted in the absence of any commercial or financial relationships that could be construed as a potential conflict of interest. The reviewer PB and the handling editor declared their shared affiliation.

## Footnotes

^1^http://dgv.tcag.ca/dgv/app/home.

^2^http://www.bioinformatics.babraham.ac.uk/projects/fastqc/.

^3^https://software.broadinstitute.org/gatk/best-practices/.

^4^http://snpeff.sourceforge.net/.

^5^www.1000genomes.org/.

^6^http://evs.gs.washington.edu/EVS/.

^7^http://www.R-project.org/.

^8^http://david.abcc.ncifcrf.gov/.
